# A clinical and pharmacokinetic study of the combination of carboplatin and paclitaxel for epithelial ovarian cancer.

**DOI:** 10.1038/bjc.1997.47

**Published:** 1997

**Authors:** N. Siddiqui, A. V. Boddy, H. D. Thomas, N. P. Bailey, L. Robson, M. J. Lind, A. H. Calvert

**Affiliations:** Nothern Centre for Cancer Treatment, Newcastle General Hospital, Newcastle upon Tyne, UK.

## Abstract

The aim of this phase I study was to determine the maximum tolerated dose of a 3-h infusion of paclitaxel, combined with carboplatin at a fixed AUC of 7 mg ml-1 min every 4 weeks for up to six cycles and to evaluate any possible pharmacokinetic interaction. Twelve chemonaive patients with ovarian cancer were treated with paclitaxel followed by a 30-min infusion of carboplatin. Paclitaxel dose was escalated from 150 mg m-2 to 225 mg m-2 in cohorts of three patients. Carboplatin dose was based on renal function. Pharmacokinetic studies were performed in nine patients (at least two at each dose level). A total of 66 courses were evaluable for assessment. Grade 3 or 4 neutropenia was seen in 70% of the courses, however hospitalization was not required. Grade 3 or 4 thrombocytopenia occurred in 24% of the courses. Alopecia, myalgia and peripheral neuropathy were common but rarely severe. The pharmacokinetics of paclitaxel was non-linear and did not appear to be influenced by co-administration of carboplatin. The AUC of carboplatin was 7.0 +/- 1.4 mg ml-1 min, indicating that there was no pharmacokinetic interaction. The combination of carboplatin and paclitaxel may be administered as first-line treatment for advanced ovarian cancer. Although myelosuppression is the dose-limiting toxicity of the component drugs, the severity of thrombocytopenia was less than anticipated. The results of this study, with only a small number of patients, need to be confirmed in future investigations.


British Journal of Cancer (1997) 75(2), 287–294
© 1997 Cancer Research Campaign

### A clinical and pharmacokinetic study of the combination of carboplatin and paclitaxel for epithelial ovarian cancer

N Siddiqui[1], AV Boddy[2], HD Thomas[2], NP Bailey[1,2], L Robson[1], MJ Lind[1,2] and AH Calvert[1,2]

[1]Northern Centre for Cancer Treatment, Newcastle General Hospital, Newcastle upon Tyne, UK; [2]Department of Oncology, University of Newcastle upon Tyne, Newcastle upon Tyne, UK

**Summary** The aim of this phase I study was to determine the maximum tolerated dose of a 3-h infusion of paclitaxel, combined with carboplatin at a fixed AUC of 7 mg ml$^{-1}$min every 4 weeks for up to six cycles and to evaluate any possible pharmacokinetic interaction. Twelve chemonaive patients with ovarian cancer were treated with paclitaxel followed by a 30-min infusion of carboplatin. Paclitaxel dose was escalated from 150 mg m$^{-2}$ to 225 mg m$^{-2}$ in cohorts of three patients. Carboplatin dose was based on renal function. Pharmacokinetic studies were performed in nine patients (at least two at each dose level). A total of 66 courses were evaluable for assessment. Grade 3 or 4 neutropenia was seen in 70% of the courses, however hospitalization was not required. Grade 3 or 4 thrombocytopenia occurred in 24% of the courses. Alopecia, myalgia and peripheral neuropathy were common but rarely severe.

The pharmacokinetics of paclitaxel was non-linear and did not appear to be influenced by co-administration of carboplatin. The AUC of carboplatin was 7.0±1.4 mg ml$^{-1}$ min, indicating that there was no pharmacokinetic interaction. The combination of carboplatin and paclitaxel may be administered as first-line treatment for advanced ovarian cancer. Although myelosuppression is the dose-limiting toxicity of the component drugs, the severity of thrombocytopenia was less than anticipated. The results of this study, with only a small number of patients, need to be confirmed in future investigations.

**Keywords:** paclitaxel; carboplatin; phase I; ovarian cancer

For the past two decades, efforts have been made to improve the treatment of ovarian cancer, but results remain far from ideal. Radical debulking surgery alone is not curative and chemotherapy is required for the majority of patients. Of the chemotherapy agents used, platinum-based regimens have proved to be the most effective (Advanced Ovarian Cancer Trial Group, 1991). These drugs, given as first-line therapy, induce responses in 50–60% of patients, but in a majority of responding patients the tumour eventually recurs (Wiltshaw et al, 1986).

One recent advance in the chemotherapy treatment of ovarian cancer has been the introduction of paclitaxel, a plant alkaloid derived from the bark of *Taxus brevifolia*, the Pacific yew tree. Paclitaxel has been evaluated in different trial settings, and response rates of 16–30% have been reported in platinum-resistant patients (Caldas and Mcguire, 1993; Trimble et al, 1993; Eisenhauer et al, 1994). These results are similar to the findings of other single-agent trials for refractory disease, with 25–40% overall response to cisplatin, one of the most effective drugs for ovarian cancer (Wiltshaw et al, 1979). This has encouraged the use of paclitaxel in combination with drugs with established activity. Cisplatin plus paclitaxel has been compared with cisplatin plus cyclophosphamide in previously untreated ovarian cancer patients. The response rates were significantly greater in the cisplatin plus paclitaxel arm with improved disease-free and overall survival (McGuire et al, 1993, 1996).

Carboplatin, an analogue of cisplatin, has proved to be equally effective in the treatment of ovarian cancer (Adams et al, 1989; Alberts et al, 1992). Unlike cisplatin, carboplatin has few non-haematological side-effects and predictable myelosuppression is its dose-limiting toxicity (Adams et al, 1989). A phase I study with a fixed dose of paclitaxel of 135 mg m$^{-2}$ given over 24 h and with escalating doses of carboplatin showed that the maximum tolerated area under the plasma concentration–time curve (AUC) of the latter drug was 7.5 mg ml$^{-1}$ min (Ozols et al, 1993).

In the present study, paclitaxel was given as a 3-h infusion immediately followed by carboplatin as a 30-min infusion. The dose of carboplatin was individualized to a target AUC of 7 mg ml$^{-1}$ min. The starting dose of paclitaxel was based on a previous report (Ozols et al, 1993), with administration over 24 h, and on the evidence that toxicity was less when the drug was administered over 3 h (Eisenhauer et al, 1994). Our aims were firstly to determine the toxicity profile of paclitaxel at escalating dose levels when combined with a standard AUC of carboplatin, secondly to establish the dose-limiting toxicity of the combination of the two drugs and, finally, to study the relationship between their pharmacokinetics and toxicity.

*Received 1 May 1996*
*Received 12 July 1996*
*Accepted 2 Aug 1996*

*Correspondence to:* A V Boddy, Department of Oncology, Cancer Research Unit, Medical School, University of Newcastle upon Tyne, Newcastle upon Tyne NE2 4HH, UK

### PATIENTS AND METHODS

### Eligibility

Twelve patients with ovarian cancer were entered into this trial between April 1994 and April 1995. All patients met the following inclusion criteria: histologically proven epithelial cancer of the ovary, chemonaive patients, aged between 18–75 years inclusive, ECOG performance status of 2, life expectancy of at least 12 weeks, absolute neutrophil count (ANC) > 1.5 × $10^9$ $l^{-1}$, platelet count > 100 × $10^9$ $l^{-1}$, total bilirubin 1.25 × upper normal limit (< 2.5 × if due to metastases), serum creatinine < 2 × upper limit of normal.

Exclusion criteria included brain metastases or leptomeningeal involvement, past or current history of other neoplasm, except curatively treated carcinoma in situ of cervix and non-melanoma skin cancer, serious cardiac disease, documented myocardial infarction within 6 months before entry, second- or third-degree heart block, active infection or history of prior allergic reaction to drugs formulated with Cremophor as an ingredient (e.g. cyclosporine or vitamin K).

Treatment was to be discontinued if patients experienced unresolved toxicity, disease progression or hypersensitivity reaction. Follow-up of disease status was performed at 3 months, 6 months and up to 2 years following study completion.

All patients provided written informed consent (approved by the local ethics committee) before starting treatment. A summary of patient characteristics and treatment is given in Table 1.

### Treatment protocol

All patients received a full clinical assessment not less than 2 weeks before entry into the sudy. With the exception of patient 6, who had only exploratory laporotomy, all patients had debulking surgery before treatment. Patient 6 had surgery after three cycles of treatment, after which the full course of chemotherapy was completed.

Paclitaxel (Taxol, Bristol-Myers Squibb) was supplied as a concentrated sterile solution of 6 mg $ml^{-1}$ in a 5-ml vial in polyoxylated castor oil (Cremophor EL) 50% and dehydrated alcohol USP 50%. The contents of the vial were diluted and administered as a continuous infusion in 500 ml of 5% dextrose/water through a peripheral line with an in-line filter. Carboplatin (Paraplatin, Bristol-Myers Squibb) was provided as lyophilized powder. Immediately before use, the drug was reconstituted with 15 ml of sterile water or 5% dextrose and diluted in 250 ml of 5% dextrose. The sequence of administration of the drugs was always paclitaxel followed by carboplatin.

All patients received 20 mg oral dexamethasone, 12 h and 6 h before treatment, and cimetidine 300 mg and chlorpheniramine 10 mg by intravenous injection, 10 min before paclitaxel infusion.

The starting dose of paclitaxel was 150 mg $m^{-2}$, increased in cohorts of three patients at each level.

| | | | |
|---|---|---|---|
| Dose level | –1 | 135 mg $m^{-2}$ | |
| Dose level | 0 | 150 mg $m^{-2}$ | patients 1–3 |
| Dose level | 1 | 175 mg $m^{-2}$ | patients 4–6 |
| Dose level | 2 | 200 mg $m^{-2}$ | patients 7–9 |
| Dose level | 3 | 225 mg $m^{-2}$ | patients 10–12 |

Up to three further patients were to be entered at a dose-level if a dose-limiting toxicity occurred. The dose of carboplatin was calculated using the Calvert formula (Calvert et al, 1989):

$$\text{Dose (mg)} = \text{target AUC (mg ml}^{-1}\text{ min)} \times (\text{GFR} + 25)$$

Glomerular filtration rate (GFR) (ml $min^{-1}$) was determined using the clearance of [$^{51}$Cr] EDTA. The target AUC of carboplatin was 7 mg $ml^{-1}$ min All doses of carboplatin were given over 30 min by intravenous infusion.

**Table 1** Patient and disease characteristics

| | |
|---|---|
| *Patient characteristics* | |
| Number of courses assessable (median per patient) | 66 (6) |
| Age in years (median) | 32–72 (58) |
| Performance status (median) | 0–2 (1) |
| *Histology* | |
| Moderately–poorly differentiated | |
| Endometrioid adenocarcinoma | 4 |
| Serous cystadenocarcinoma | 3 |
| Clear cell carcinoma | 2 |
| Mucinous adenocarcinoma | 1 |
| Adenocarcinoma | 1 |
| Poorly differentiated | |
| Adenocarcinoma | 1 |
| Stage | |
| IIb 1 | |
| IIc 3 | |
| III 6 | |
| IV 2 | |
| Residual disease (CT abdomen and pelvis) | |
| None 6 | |
| Minimal | 1 |
| 2–5 cm | 2 |
| >5 cm | 1 |
| Liver metastases | 2 |

The above schedule was to be modified according to haematological toxicity. Dose reduction was to be performed if ANC dropped below 0.5 × $10^9$ $l^{-1}$ and lasted for 7 days or more, or if grade 4 thrombocytopenia occurred requiring platelet transfusion. The chemotherapy cycles were given at 4-weekly intervals. Granulocyte colony stimulating factor (GCSF) was to be administered only if clinically indicated. Nausea and vomiting were controlled with ondansetron.

### Evaluation of toxicity and response

Toxicity was assessed for each cycle using WHO criteria. Weekly blood counts were performed to evaluate the haematological and biochemical profiles. Physical examination was carried out on day 1 of each cycle. Tumour measurements (when applicable) were made every cycle by physical examination, every month by CA 125 and every three cycles by computerized tomography (CT) scans. Clinical response of the tumour was assessed according to UICC criteria.

Although this was a dose-finding study, patients with evaluable disease were assessed for response, using the following criteria:

| | |
|---|---|
| Complete response (CR) | Disappearance of all clinical evidence of tumour, determined by two observations not less than 4 weeks apart. |
| Partial response (PR) | 50% or greater decrease in the sum of products of measured indicator lesions with no simultaneous increase in the size of any lesion or appearance of new lesions. |
| Stable disease (SD) | Response less than 50%, steady state of response or progression less than progressive disease (PD). |

© Cancer Research Campaign 1997

Progressive disease (PD) Unequivocal increase of at least 25% in the product of measured lesions.

Marker responses were determined as follows:

CR Complete normalization of CA 125, lasting for more than 4 weeks.
PR Decrease of CA 125 levels to less than 50% of pretreatment level for more than 4 weeks.
SD Less than 50% reduction or less than 25% increase in CA 125 levels.
PD More than 50% increase in CA 125 levels.
NE Pretreatment level is normal (< 35 $kUL^{-1}$) or less than 100 $kUL^{-1}$.

The maximum tolerated dose was defined as that which caused ANC less than $0.5 \times 10^9$ $l^{-1}$ lasting more than 7 days, neutropenic sepsis, grade 4 thrombocytopenia, non-haematological toxicity (Grade 3 or greater) or absence of recovery from toxicity at scheduled retreatment in three or more of six patients treated at that dose level.

### Pharmacokinetic studies

*Methods*

Of the 12 patients enrolled in the study, nine were willing and able to undergo collection of plasma for pharmacokinetic analysis. Samples of blood (10 ml) were taken at times before, during and up to 19 h after infusion of paclitaxel and anticoagulated with EDTA. Plasma was separated immediately and stored at –20°C until analysis. Urine was collected and stored at –20°C or was treated with Cremophor/ethanol to give a final concentration of solubilizing agents of 10%.

Samples and standards were analysed by an HPLC method similar to that described by Huizing et al (1993). Briefly, standards of paclitaxel in plasma (0.02–10 μg $ml^{-1}$) were prepared by serial dilution of a stock solution in methanol. A 0.5-ml aliquot of each sample or standard was mixed with 0.5 ml of 0.02 M ammonium acetate buffer (pH 5.0) and centrifuged at 1000 r.p.m. for 10 min before extracting. Samples were extracted using end-capped cyano Isolute columns (IST, Mid-Glamorgan, UK), preconditioned with 2 ml of methanol followed by 2 ml of 0.02 M ammonium acetate buffer (pH 5.0). After application of the buffered sample, the column was washed with 4 ml of buffer followed by 2 ml of 20% methanol in buffer and 1 ml of isohexane. Analytes were eluted into clean tubes using 2 ml of 0.1% triethylamine in acetonitrile. The eluant was evaporated to dryness under nitrogen at 30°C. Samples were reconstituted in 200 μl of 50% acetonitrile/buffer mixture, mixing each sample for 20 sec on a vortex mixer.

The HPLC system consisted of a Waters pump (Milford, MA, USA) at 1.0 ml $min^{-1}$, a Pye Unicam PU 4021 dual wavelength UV detector (Cambridge, UK) at 227 and 235 nm. The column was a 250 × 4.6 mm APEX $C_6$ 5-μ column (Jones, Mid-Glamorgan, UK), protected with a pelicular $C_{18}$ precolumn. The mobile phase was a 50% mixture of acetonitrile and 0.02 M ammonium acetate buffer (pH 5.0).

Urine was analysed as for plasma except that buffered samples (1 ml) were extracted into 1-chlorobutane (5 ml), the organic phase separated and the solvent evaporated before reconstitution and chromatography.

Total platinum was determined in samples taken 24 h after the start of administration using flameless atomic absorption spectrophotometry (Harland et al, 1984). This was used to calculate the free platinum AUC using a previously published method (Ghazal-Aswad et al, 1996).

The pharmacokinetics of paclitaxel was determined by model-independent analysis with clearance and volume of distribution at steady-state estimated by moment analysis (Gibaldi and Perrier, 1982). Terminal half-life was estimated by log-linear regression of the last four data points. In addition, a two-compartment model was fitted to each data set using Adapt II, release 3 (D'Argenio and Schumitsky, 1990). Parameter estimates for each individual were used to calculate durations for which plasma concentrations of paclitaxel exceeded thresholds for pharmacological effect of 0.05 μM (Gianni et al, 1995) and 0.1 μM (Huizing et al, 1995).

### RESULTS

A total of 12 patients received 66 courses at four dose levels. One patient was withdrawn from the study because of peripheral neuropathy. A second patient, who had presented with stage IV disease, had evidence of progression after the second cycle of treatment and was withdrawn from study.

### Analysis of toxicity

Overall, the treatment was well tolerated. Ten patients completed the treatment of six cycles each.

*Haematological toxicity*

The major haematological toxicity observed was neutropenia (Table 2). Nadir neutrophil counts were most commonly seen at day 14, with some degree of recovery by day 21. Nineteen per cent of courses resulted in neutrophil nadirs less than $0.5 \times 10^9$ $l^{-1}$ (Grade 4). Neutropenia was most marked in patients treated at the highest dose level (Table 2). Despite the degree of neutropenia, none of the patients developed any consequent clinical problems. Administration of GCSF was not necessary in any case. Treatment had to be deferred by 1 week in one patient (dose level 2, course 5) as a result of leucopenia on the day of treatment (WBC $2.3 \times 10^9$ $l^{-1}$).

The nadir for platelets was also seen at day 14. Thrombocytopenia (WHO grade 3 or 4) was seen in 5 out of 16 courses at dose level 0, and one dose level reduction was required for patient 3. However, at a dose of paclitaxel of 175 mg $m^{-2}$, a significant drop in platelets was seen in only 1 of 18 courses. The drop in platelet count was least at this dose level (Table 2). Two patients required platelet transfusions, one on two occasions.

It should be noted that some patients consistently developed grade 3 or 4 haematological toxicity throughout their treatment, while others at the same dose level showed only minimal toxicity. Anaemia was not a major problem.

*Non-haematological toxicity*

Alopecia was seen in all patients and was total. The majority lost their hair by course 3 of treatment. The onset of hair loss was quite sudden in some patients. Peripheral neuropathy was the other predominant toxicity. Patient 1, who was withdrawn from the study, developed tingling in the fingers of both hands after the first course of treatment. By the time four courses were completed, symptoms had progressed, and paraesthesia had developed with numbness in fingers and toes. On examination, there was a reduction in sensations

© Cancer Research Campaign 1997

**Table 2** Haematological toxicities of carboplatin plus escalating doses of paclitaxel: numbers of courses at each toxicity grade

| Dose level | Patient no. | Total no. of courses | Neutropenia (WHO grade) | | | | | Thrombocytopenia (WHO grade) | | | | | Comments |
|---|---|---|---|---|---|---|---|---|---|---|---|---|---|
| | | | 0 | 1 | 2 | 3 | 4 | 0 | 1 | 2 | 3 | 4 | |
| 0.150 mg m-2 | 1 | 4 | 0 | 1 | 2 | 1 | 0 | 1 | 0 | 3 | 0 | 0 | |
| | 2 | 6 | 0 | 0 | 0 | 6 | 0 | 3 | 2 | 0 | 1 | 0 | |
| | 3 | 6 | 0 | 1 | 1 | 4 | 0 | 0 | 0 | 2 | 3 | 1 | (Platelet transfusion × 1) DR |
| 1.175 mg m-2 | 4 | 6 | 0 | 0 | 1 | 3 | 2 | 2 | 0 | 3 | 1 | 0 | |
| | 5 | 6 | 0 | 1 | 3 | 2 | 0 | 6 | 0 | 0 | 0 | 0 | |
| | 6 | 6 | 0 | 0 | 0 | 5 | 1 | 5 | 0 | 1 | 0 | 0 | |
| 2.200 mg m-2 | 7 | 2 | 1 | 0 | 1 | 0 | 0 | 2 | 0 | 0 | 0 | 0 | |
| | 8 | 6 | 0 | 0 | 1 | 4 | 1 | 1 | 0 | 1 | 1 | 3 | (Platelet transfusion × 3) DR |
| | 9 | 6 | 0 | 1 | 2 | 1 | 2 | 2 | 3 | 1 | 0 | 0 | |
| 3.225 mg m-2 | 10 | 6 | 0 | 0 | 0 | 1 | 5 | 2 | 3 | 1 | 0 | 0 | |
| | 11 | 6 | 0 | 0 | 0 | 3 | 3 | 0 | 0 | 1 | 2 | 3 | |
| | 12 | 6 | 0 | 0 | 1 | 3 | 2 | 1 | 1 | 3 | 1 | 0 | |

DR dose reduction.

**Figure 1** Overall incidence of non-haematological toxicities of paclitaxel in combination with a fixed AUC of carboplatin

**Figure 2** Calculated AUC of carboplatin at different dose levels of paclitaxel. Dotted line shows target AUC

**Figure 3** Clearance of paclitaxel at different dose levels and hyporeflexia. All other patients had paraesthesias, but few had numbness of fingers and toes. Subjective symptoms of peripheral neuropathy were more frequent than would be suggested by physical examination. Neurotoxicity appeared to be cumulative, but was not related to dose.

Three patients complained of nausea and vomiting (Figure 1) which was controlled with ondansetron, however one of these (patient 12) had more severe (Grade 3) vomiting, requiring the addition of lorazepam and prochlorperazine. All except two patients had mild myalgia from day 2 to 6 during the first week after treatment. This was relieved by non-steroidal anti-inflammatory drugs. Cardiotoxicity as such was not seen. Two patients, who were known to be hypertensive before starting treatment, complained of mild dizziness for a few days after treatment. One patient (patient 8) developed a pulmonary embolism and needed anticoagulants, another (patient 10) suffered a deep vein thrombosis of the leg. Other toxicities were mild and only occasionally encountered, e.g. mucositis, constipation, diarrhoea and hypertension. As all the patients received prophylactic medication, no hypersensitivity reaction attributable to paclitaxel was observed. No hepatic or renal toxicity was encountered.

### Analysis of pharmacokinetics

### *Carboplatin pharmacokinetics*

The AUC of free platinum was estimated in each patient using the total platinum in a sample taken 24 h after administration. In the nine patients for whom plasma samples were available, the estimated AUC was, on average, equal to the target AUC, but with some variation (mean ± s.d.; 7.0±1.4 mg $ml^{-1}$ min). There did not

*© Cancer Research Campaign 1997*

**Table 3** Pharmacokinetics of paclitaxel and its 6-hydroxy metabolite when administered in combination with carboplatin

| Patient | Surface area (m$^{-2}$) | Paclitaxel dose (mg m$^{-2}$) | Peak level (µg ml$^{-1}$) | AUC (µg ml$^{-1}$ min) | Half-life (min) | $V_{dss}$ (l m$^{-2}$) | Cl (ml min$^{-1}$ m$^{-2}$) | Excretion in urine (% of dose) | AUC 6-hydroxy metabolite (µg ml$^{-1}$ min) |
|---|---|---|---|---|---|---|---|---|---|
| 1 | 1.8 | 150 | 2.6 | 481 | 408 | 74 | 312 | 3.6 | 26 |
| 2 | 2.1 | 150 | 2.9 | 507 | 470 | 78 | 296 | 3.3 | 20 |
| 3 | 1.7 | 150 | 2.8 | 439 | 602 | 113 | 342 | 5.8 | 16 |
| 4 | 1.5 | 175 | 3.2 | 607 | 366 | 68 | 288 | 3.6 | 14 |
| 5 | 1.9 | 175 | 4.5 | 772 | 669 | 90 | 227 | 1.9 | 33 |
| 8 | 1.5 | 200 | 7.7 | 1489 | 503 | 61 | 134 | 2.9 | 52 |
| 9 | 1.7 | 200 | 7.1 | 1433 | 343 | 28 | 141 | 5.4 | 46 |
| 10 | 1.6 | 225 | 6.9 | 1787 | 361 | 38 | 126 | 3.4 | 106 |
| 12 | 1.7 | 225 | 7.6 | 1569 | 384 | 30 | 143 | 3.2 | 113 |

$V_{dss}$, volume of distribution at steady state; Cl, clearance.

**Figure 4** Plasma concentrations of paclitaxel and its 6-hydroxy metabolite in patient 10 (dose 225 mg m$^{-2}$). ■ Paclitaxel; ▲ 6-hydroxypaclitaxel

**Figure 5** Graph of percentage decrease in ANC against time for which paclitaxel concentrations in plasma exceed 0.05 µM (◆) or 0.1 µM (□)

**Table 4** Response to carboplatin in combination with escalating doses of paclitaxel – Tumour marker levels (CA 125)

| Dose level | Patient no. | CA125 (kU l$^{-1}$) Pretreatment[a] | Post-treatment[b] |
|---|---|---|---|
| 0.150 mg m$^{-2}$ | 1 | 240 | 9 |
| | 2 | 115 | 18 |
| | 3 | 102 | 27 |
| 1.175 mg m$^{-2}$ | 4 | 46 | 40 |
| | 5 | 67 | 18 |
| | 6 | 540 | 8 |
| 2.200 mg m$^{-2}$ | 7 | 171 | 108 |
| | 8 | 17 | 8 |
| | 9 | 197 | 7 |
| 3.225 mg m$^{-2}$ | 10 | 550 | 31 |
| | 11 | 221 | 21 |
| | 12 | 195 | 9 |

[a]Within 2 weeks of entering trial. [b]After completion of up to six courses of therapy.

appear to be any influence of paclitaxel dose on the AUC of free platinum (Figure 2).

### *Paclitaxel pharmacokinetics:*

As for carboplatin, plasma samples were available for only nine patients. Paclitaxel reached a maximum plasma concentration (2.6–7.7 µg ml$^{-1}$) at the end of the infusion and was still detectable in every patient 24 h later (0.02–0.08 µg ml$^{-1}$). Although a linear two-compartment model appeared to be adequate to describe the pharmacokinetics of paclitaxel in individual patients (data not shown), comparison of clearance at the different dose levels revealed obvious non-linearity in elimination over this dose range (Figure 3). For a 33% increase in dose from 150 to 200 mg m$^{-2}$, the AUC increased threefold (mean 476 µg ml$^{-1}$ min$^{-1}$ to 1461 µg ml$^{-1}$ min). Volume of distribution appeared to be dose dependent (Table 3), but calculation of this parameter is not reliable in the presence of non-linear elimination. Terminal half-life did not appear to be dose-dependent and varied from 5.7 h up to 11.1 h (Table 3).

Only a small fraction (< 6%) of the administered dose was collected in the urine as unchanged paclitaxel (Table 3). Metabolite peaks were noted in chromatograms from both plasma and urine and were tentatively identified, by comparison with published retention times (Huizing et al, 1993), as the 6-hydroxy and the 3′-phenyl-hydroxy metabolites described previously. Approximate AUC values for the 6-hydroxy metabolite are given in Table 3. A plot of plasma concentration against time for the parent drug and the 6-hydroxy metabolite in one patient is shown in Figure 4.

The durations for which paclitaxel concentrations exceeded previously identified thresholds for pharmacological effect were calculated. The duration above 0.1 µM ranged from 775 to 1665 min, while that above 0.05 µM went from 1125 to 2115 min. There were clear differences among patients in the rank order of the duration above these two threshold concentrations; and these were due to interindividual variations in the elimination rates post-infusion. There was no clear relationship between toxicity and time above these threshold concentrations (Figure 5). This may be because only a narrow range of toxicities was observed in only a small number of patients.

### Analysis of response

All of the patients were assessed for response by clinical, biochemical and radiological parameters. In many studies, serum CA 125 tumour marker level has been considered a reliable predictor of response to treatment (McGuire et al, 1989; Einzig et al 1992; Kohn et al, 1994; Tuxen et al, 1995). Of the twelve patients who entered the study, nine patients had pretreatment CA 125 levels of ≥100 kU $l^{-1}$ (normal range: up to 35 kU $l^{-1}$). In all except one of these patients, the levels normalized during treatment (Table 4).

Six patients had normal base line CT scans. One of these patients, who had umbilical metastases at presentation and who could not have complete debulking at the time of original surgery, progressed and was taken off the study after two courses. In six patients, residual disease was documented by CT scans before starting treatment. CT scans at the end of treatment showed CR in five of the patients with residual disease. Patient 6 had debulking surgery after course 3, before completing the final three cycles of treatment. At laproscopy, after finishing treatment, there was no evidence of disease.

Despite an initial response, six patients have relapsed since finishing chemotherapy. Two of these patients were treated at the first dose level (150 mg $m^{-2}$). Patient 1, who relapsed 9 months after her last treatment, had been withdrawn from the study because of neurotoxicity. After four courses of the combined regimen, this patient also received two further courses of single agent carboplatin (AUC 7 mg $ml^{-1}$min). Patients 2, 4, 6, 10 and 12 relapsed between 2 and 16 months after starting treatment. At present six patients remain disease free at between 15 and 24 months after the start of treatment.

### DISCUSSION

Until recently, chemotherapy with platinum regimens has been the treatment of choice for ovarian cancer. However, since the discovery of paclitaxel there has been considerable enthusiasm for combination chemotherapy based on paclitaxel with other agents (Kohn et al, 1993; Bruckner et al, 1994). The response rates and toxicity profiles of paclitaxel and cisplatin have been assessed (Rowinsky et al, 1991). The first combination of paclitaxel and cisplatin showed that there were clinically significant interactions between the two drugs (Rowinsky et al, 1991). Different schedules of drug sequences demonstrated more profound myelosuppression when cisplatin was given before paclitaxel. Neutropenia was the dose-limiting toxicity at doses of cisplatin of 75 mg $m^{-2}$ and paclitaxel of 135 mg $m^{-2}$. In a later study when GCSF (granulocyte colony-stimulating factor) was used, the dose of paclitaxel could be escalated to 250 mg $m^{-2}$ with cisplatin at 75 mg $m^{-2}$, and neuropathy became the major toxicity (Rowinsky et al, 1993). Cisplatin is known to be neurotoxic and the effects of paclitaxel on the neuromuscular system are well documented (Sarosy et al, 1992). A 3-h infusion of paclitaxel administered to patients previously treated with cisplatin induced moderate neurotoxicity and pathologically proven axonal damage (Cavaletti et al, 1995). This indicates that damage to the neurological system could be a potential problem with the combination of paclitaxel and cisplatin.

Paclitaxel is also being studied in combination with carboplatin (Ozols et al, 1993; Bolis et al, 1995; Bookman et al, 1995; Lhomme et al, 1995), but the toxicity profile of these drugs given together has yet to be properly defined. Previous phase I studies of carboplatin and paclitaxel have used the Jellife formula or creatinine clearance to determine the GFR and to calculate the dose of carboplatin. The present study is one of the first of this combination, in which the dose of carboplatin was calculated using [$^{51}$Cr]EDTA clearance to determine the glomerular filtration rate (GFR), and it would be expected to produce a very predictable level of thrombocytopenia (Calvert, 1994).

The degree of thrombocytopenia induced by the two drugs together was less than would have been expected if carboplatin had been given alone. This interesting observation, which has also been made by other groups (Bunn and Kelly, 1995; Kearns et al, 1995), is suggestive of a protective effect of paclitaxel on platelets. The mechanism underlying this observation is not yet clear, however there is some evidence that paclitaxel, like lipopolysaccharides, causes increased expression of the cytokine interleukin 1 in paclitaxel-activated macrophages (Carboni et al, 1993; O'Brien et al, 1995). It is possible that, by stimulation of cytokine production, paclitaxel causes increased levels of thrombopoieitin, thus ameliorating the degree of thrombocytopenia. Another possible mechanism for the protection of platelets is the very high degree of binding of paclitaxel to tubulin in these cells (Wild et al, 1995). This binding might, in some way, stabilize the thrombocytes (Crook and Crawford, 1989) and either protect them from platinum-induced damage or prolong circulation time.

The effect of the sequence of drug administration on haematological toxicity have been investigated (Rowinsky et al, 1991). In a study of cisplatin (50 or 75 mg $m^{-2}$) combined with paclitaxel (110–200 mg $m^{-2}$ as a 24-h infusion) and with the order of administration randomized, more profound myelosuppression was observed when cisplatin was given before paclitaxel. Pharmacological studies showed that paclitaxel clearance was 25% lower with this sequence of drugs, which may partly explain this clinically significant interaction. No such sequence-dependent interaction has been reported with carboplatin plus paclitaxel, but this has not yet been investigated systematically.

A large study, designed specifically to determine the optimal dose and schedule for paclitaxel administration, has shown that a 3-h infusion is less myelosuppressive than a 24-h infusion and that a dose of 175 mg $m^{-2}$ produces a greater anti-tumour effect (Eisenhauer et al, 1994). As a result of this, paclitaxel (as a single agent) is usually given at a dose of 175 mg $m^{-2}$ over 3 h. In this phase I study, paclitaxel administered over 3 h, in combination with carboplatin, was dose escalated from 150 to 225 mg $m^{-2}$. The maximum tolerated dose (MTD) was defined as the dose at which three or more patients developed grade 4 neutropenia or thrombocytopenia lasting for more than 7 days. This level of toxicity was not encountered, and so the MTD was not determined in this study.

It is encouraging to note that although the toxicity of this combination is low, it has been reported that the majority of patients show some tumour response (Ozols et al, 1993). The promising response rates may be owing to the fact that the response to single-agent carboplatin treatment is strongly related to the expression of mutant or wild type *p53* gene in the tumour. Although ovarian cancer cell lines with mutant *p53* genes undergo apotosis in response to both paclitaxel and cisplatin (Havrilesky et al, 1995), platinum-resistant tumours are more likely to have evidence of *p53* mutation (Al-Azraqi et al, 1995). Preclinical evidence

 © Cancer Research Campaign 1997

suggests that paclitaxel may produce apoptosis by a *p53*-independent mechanism (Woods et al, 1995). Thus, this combination of two drugs with complementary activity could prove advantageous in the treatment of a tumour cell population with heterogeneous *p53* expression.

Although not definite, there may be a relationship between paclitaxel dose and anti-tumour activity (Sarosy et al, 1992; Nabboltz et al, 1993; Belani et al, 1995; Bunn and Kelly, 1995). Non-linear pharmacokinetics could partly explain this finding. If such a dose – response effect is clearly established, it would be logical to give higher doses of paclitaxel with the support of growth factors. In a recent review of treatment for ovarian cancer, it was recommended that the standard first-line chemotherapy for advanced ovarian carcinoma should be a combination of paclitaxel plus a platinum compound (Thigpen et al, 1994). Our study, albeit with only a small number of patients, suggests that paclitaxel plus carboplatin may have an advantage over the combination of paclitaxel and cisplatin in terms of toxicity profile. Further studies with increased dose intensity, possibly supported with GCSF, are warranted, with close monitoring of the pharmacology and toxicity of both agents.

### REFERENCES

Adams M, Kerby I J, Rocker I, Evans A, Johansen K and Franks C R (1989) A comparison of the toxicity and efficacy of cisplatin and carboplatin in advanced ovarian cancer. *Acta Oncologica* **28**: 57–60

Advanced Ovarian Cancer Trial Group (1991) Chemotherapy in advanced ovarian cancer: an overview of randomised clinical trials. *Br Med J* **303**: 884–893

Al-Azraqi, A, Chapman C, Challen C, Aswad S, Sinha D, Calvert A and Lunec J (1995) P53 mutations in primary human ovarian cancer as a determinant of resistance to carboplatin. *Proc Am Assoc Cancer Res* **36**: 228

Alberts D S, Green S, Hannigan E V, O'Toole R, Stock-Novack D, Anderson P, Surwit E A, Malvya V K, Nahhas W A and Jolles CJ (1992) Improved therapeutic index of carboplatin plus cyclophosphamide versus cisplatin plus cyclophosphamide: final report by the Southwest Oncology Group of a Phase III randomized trial in Stages III and IV ovarian cancer. *J Clin Oncol* **10**: 706–717

Belani C P, Aisner J, Hiponia D and Engstrom C (1995) Paclitaxel and carboplatin with and without Filgastrim support in patients with metastatic non-small cell lung cancer. *Semin Oncol* **22** (suppl.9): 7–12

Bolis G, Scarfone G, Maggi R, Melpignanao M, Parazzini F, Presti M, Zanaboni F, Villa A and Gentile A (1995) A dose finding study with fixed dose of carboplatin and escalating paclitaxel (PTX) in advanced ovarian cancer. *Proc Am Soc Clin Oncol* **14**: 270

Bookman M A, McGuire W P, Kilpatrick E, Keenan E, Johnson S, O'Dwyer P, Rowinskky E and Ozols, R F (1995) Phase I gynecologic-oncology group (GOG) study of 3-H and 24-H paclitaxel with carboplatin as initial therapy for advanced epithelial ovarian cancer (OvCa). *Proc Am Soc Clin Oncol* **14**: 271

Bruckner H W, Cagnoni P J, Lee J-M, Chesser M R and Andreotti P E (1994) A sequence of adriamycin and taxol infusions for refractory ovarian cancer. *Proc Am Soc Clin Oncol* **13**: 276 (890)

Bunn P A and Kelly K A (1995) A Phase I study of carboplatin and paclitaxel in non-small cell lung cancer: a University of Colorado Cancer Center study. *Semin Oncol* **22** (suppl. 9): 2–6

Caldas C and Mcguire W P (1993) Paclitaxel (Taxol) therapy in ovarian carcinoma. *Semin Oncol* **20**: 50–55

Calvert A H (1994) Dose optimisation of carboplatin in adults. *Anticancer Res* **14**: 2273–2278

Calvert A H, Newell D R, Gumbrell L A, O'Reilly S, Burnell M, Boxall F E, Siddik Z H, Judson I R, Gore M E and Wiltshaw E (1989) Carboplatin dosage: prospective evaluation of a simple formula based on renal function. *J Clin Oncol* **7**: 1748–1756

Carboni J M, Singh C and Tepper M A (1993) Taxol and lipopolysaccharide activation of a macrophage cell line induction of similar tyrosine phosphoproteins. *J Nat Cancer Inst Monogr.* **15**: 95–101

Cavaletti G, Boglilun G, Marzorat L, Zincone A, Marzola M, Colombo N and Tredici G (1995) Peripheral neurotoxicity of taxol in patients previously treated with cisplatin. *Cancer* **75**: 1141–1150

Crook M and Crawford N (1989) Electrokinetic, analytical and functional heterogeneity of circulating human platelets: separation of subpopulations by continuous flow electrophoresis after taxol stabilization. *Biochim Biophy Acta – Mol Cell Res* **1014**: 26–39

D'Argenio D Z and Schumitsky A (1990) *Adapt II. Interactive Mathematical Software for Pharmacokinetic/Pharmacodynamic Systems Analysis.* Biomedical Resources, University of Southern California: Los Angeles.

Einzig A I, Wiernik P H, Sasloff J, Runowizc C D and Goldberg G L (1992) Phase II study and long term follow-up of patients treated with taxol for advanced ovarian adenocarcinoma. *J Clin Oncol* **10**: 1748–1753

Eisenhauer E A, ten Bokkel Huinik W W, Swenerton K D, Gianni L, Myles J, van der Burg MEL, Kerr I, Bermorken J B, Buser K, Colombo N, Bacon M, Santabarbara P, Onetto N, Winograd B and Canetta R (1994) European–Candian randomized trial of paclitaxel in relapsed ovarian cancer: high-dose versus low-dose and long versus short infusion. *J Clin Oncol* **12**: 2654–2666

Ghazal-Aswad S, Calvert A H and Newell D R (1996) A single sample assay for the calculation of the area under the free carboplatin plasma concentration versus time curve. *Cancer Chemother Pharmacol* **37**: 429–434

Gianni L, Kearns C, Giani A, Capri G, Vigano L, Locatelli A, Bonadonna G and Egorin M (1995) Nonlinear pharmacokinetics and metabolism of paclitaxel and its pharmacokinetic/pharmacodynamic relationships in humans. *J Clin Oncol* **13**: 180–190

Gibaldi M and Perrier D (1982) Pharmacokinetics, Vol. 15. Marcel Dekker: New York.

Harland S J, Newell D R, Siddik Z H, Chadwick R, Calvert A H and Harrap K R (1984) Pharmacokinetics of cis-diammine-1,1-cyclobutane dicarboxylate platinum(II) in patients with normal and impaired renal function. *Cancer Res* **44**: 1693–1697

Harvilesky L J, Elbendary A, Hurteua J A, Whitaker R S, Rodriguez G C and Berchuck A (1995) Chemotherapy-induced apoptosis in epithelial ovarian cancers. *Obstet Gynecol* **85**: 1007–1010

Huizing M T, Keung ACF, Rosing H, van der Kuij V, ten Bokkel-Huinink W W, Mandjes I M, Dubbelman ARC. Pinedo H M and Beijnen J H (1993) Pharmacokinetics of paclitaxel and metabolism in a randomised comparative study in platinum-pretreated ovarian cancer patients. *J Clin Oncol* **11**: 2127–2135

Huizing M T, van Warmerdam LJC, Rosing H, ten Bokkel Huinink W W, Stewart M B, Pinedo H M and Beijnen J H (1995) Limited sampling strategies for investigating paclitaxel pharmacokinetics in patients receiving 175 mg/m$^2$ as a 3-hour infusion. *Clin Drug Invest* **9**: 344–353

Kearns C M, Belani C P, Erkmen K, Zuhowski M, Hiponia D, Engstrom C, Ramanathan R, Trenn M and Egorin M J (1995) Reduced platelet toxicity with combination carboplatin and paclitaxel: pharmacodynamic modulation of carboplatin associated thrombocytopenia. *Proceedings of the International Symposium on Platinum and other Metal Compounds in Cancer Chemotherapy*, Amsterdam. Abstract 070. Plenum Press: New York

Kohn E, Reed E, Link C, Christian M, Davis P and Sarosy G (1993) A pilot study of taxol, cisplatin, cyclophosphamide, and G-CSF in newly diagnosed stage III/IV ovarian cancer. *Proc Am Soc Clin Oncol* **12**: 257 (814).

Kohn E C, Sarosy G, Bicher A, Link C, Christian M, Steinberg S M, Rothenberg M, Adamo D O, Davis P, Ognibene F P, Cunnion R E and Reed E (1994) Dose-intense taxol: high response rate in patients with platinum-resistant recurrent ovarian cancer. *J Nat Cancer Inst* **86**: 18–24

Lhomme C, Kerbrat P and Guastalla J P (1995) Phase I escalating dose of Taxol (T) (paclitaxel) over 3H in combination with carboplatin (Cb) 400 mg/m$^2$ as first line chemotherapy (CT) in ovarian cancer (OC). *Proc Am Soc Clin Oncol* **14**: 798

McGuire W P, Hoskin W J, Brady M F, Kucera P R, Look K Y, Partridge E E and Davidson M (1993) A Phase III trial comparing cisplatin/cytoxan (PC) and cisplatin/taxol (PT) in advanced ovarian cancer (AOC). *Proc Am Soc Clin Oncol* **12**: 255 (808)

McGuire W P, Hoskins W J Brady M F, Kucera P R, Partridge E E, Look K Y, Clarke-Pearson D L and Davidson M (1996) Cyclophosphamide and cisplatin compared with paclitaxel and cisplatin in patients with stage III and stage IV ovarian cancer. *New Engl J Med* **334**: 1–7

McGuire W P, Rowinsky E K, Rosenshein N B, Grumbine F C, Ettinger D S, Armstrong D K and Donehower R C (1989) Taxol (T): a unique antineoplastic agent with significant activity in advanced ovarian epithelial neoplasms. *Ann Inter Med* **111**: 273–279

Nabboltz J M, Gelmon K, Bontenbal M, Spielmann M, Clavel M, Seeber S, Conte P, Namer M, Bonneterre J, Fumoleau P, Sulkes A, Sauter C, Roche H, Calvert H, Kaufmann J, Chazard M, Diergarten K, Gallant G, Thompson M,

Winograd B and Onetto N (1993) Randomized trial of Taxol in metastatic breast cancer: an interim analysis. *Proc Am Soc Clin Oncol* **12**: 60 (42)

O'Brien J M, Wewers M D, Moore S A and Allen J N (1995) Taxol and colchicine increase LPS-induced Pro-IL-1b production, but do not increase IL-1b secretion. *J Immunol* **154**: 4113–4222

Ozols R F, Kilpatrick D, O'Dwyer P, Hohnson S, Bookman M A, Walczak J, Rowinsky E and McGuire W P (1993) Phase I and pharmacokinetic study of paclitaxel (T) and carboplatin (C) in previously untreated patients (PTS) with advanced epithelial ovarian cancer (OC): a pilot study of the Gynaecologic Oncology Group. *Proc Am Soc Clin Oncol* **12**: 259 (824)

Rowinsky E K, Chaudhry V, Forastiere A A, Sartoius S E, Ettinger D S, Grochow L B, Lubejko B G, Cornblath D R and Donehower R C (1993) Phase I and pharmacologic study of paclitaxel and cisplatin with granulocyte colony stimulating factor: neuromuscular toxicity is dose limiting. *J Clin Oncol* **11**: 2010–2020

Rowinsky E K, Gilbert M R, McGuire W P, Noe D A, Grochow L B, Forastiere A A, Ettinger D S, Lubejko B G, Clark B, Sartorius S E, Cornblath D R, Hendricks C B and Donehower R C (1991) Sequences of taxol and cisplatin: a phase I and pharmacologic study. *J Clin Oncol* **9**: 1692–1703

Sarosy G, Kohn E, Stone D A, Rothenberg M, Jacob J, Adamo D O, Ognibene F P, Cunnion R E and Reed E (1992) Phase I study of taxol and granulocyte colony stimulating factor in patients with refractory ovarian cancer. *J Clin Oncol* **10**: 1165–1170

Thigpen T, Vance R, Puneky L and Khansur T (1994) Chemotherapy in advanced ovarian carcinoma: current standards of care based on randomized trials. *Gynecol Oncol* **55**: S97–S107

Trimble E L, Adams J D, Vena D, Hawkins M J, Friedman M A, Fisherman J S, Christian M C, Canetta R, Onetto N, Hayn R and Arbuck S G (1993) Paclitaxel for platinum refractory ovarian cancer: results from the first 1,000 patients registered to National Cancer Institute Treatment Referral Center 9103. *J Clin Oncol* **11**: 2405–2410

Tuxen M K, Soletormow G and Dombernowsky P (1995) Tumour markers in the management of patients with ovarian cancer. *Cancer Treat Rev* **21**: 215–245

Wild M D, Walle U K and Walle T (1995) Extensive and saturable accumulation of paclitaxel by the human platelet. *Cancer Chemother Pharmacol* **36**: 41–44

Wiltshaw E, Evans B, Rustin G, Gilbey E, Baker J and Barker G (1986) A prospective randomized trial comparing high-dose cisplatin with low-dose cisplatin and chlorambucil in advanced ovarian carcinoma. *J Clin Oncol* **4**: 772–729

Wiltshaw E, Subramarian S, Alexopoulos C and Barker GH (1979) Cancer of the ovary: a summary of experience with cis-dichlorodiamminoplatinum(II) at the Royal Marsden Hospital. *Cancer Treat Reports* **63**: 1545–1548

Woods C M, Zhu J, McQueney P A, Bollag D and Lazarides E (1995) Taxol-induced mitotic block triggers rapid onset of a p53-independent apoptotic pathway. *Molecular Medicine* **1**: 506–526

*© Cancer Research Campaign 1997*


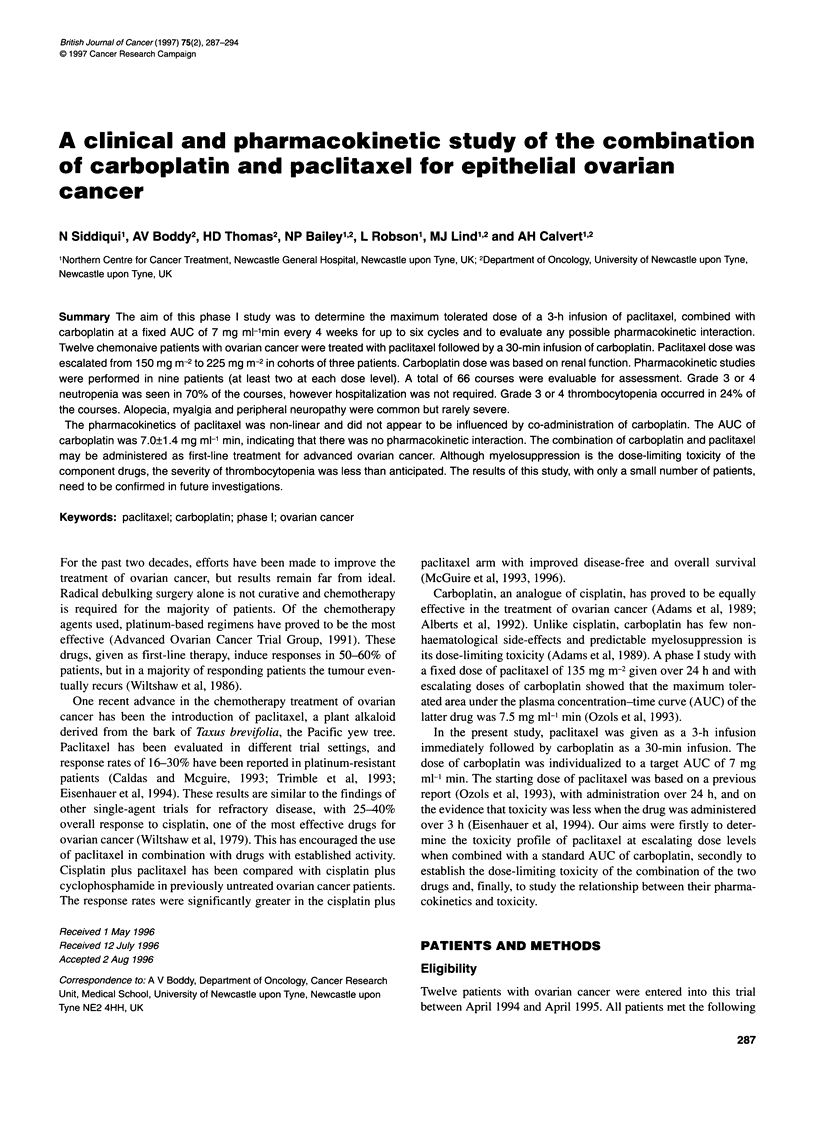

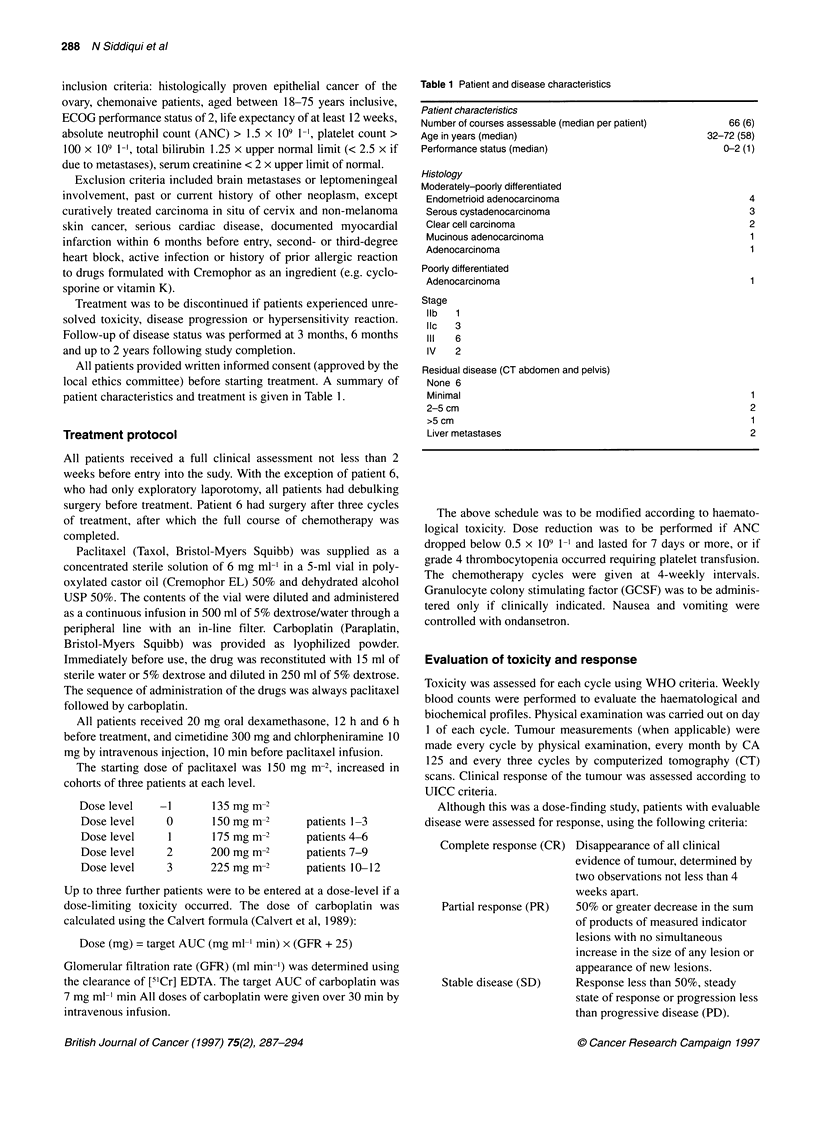

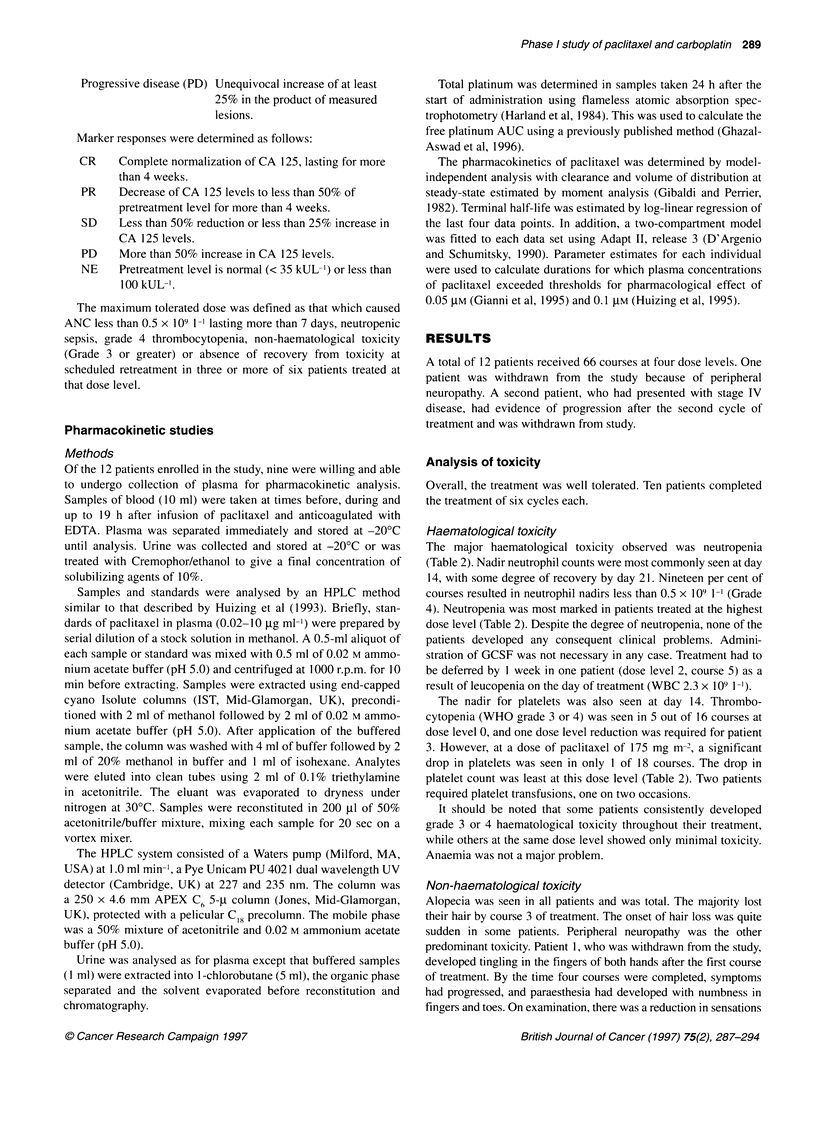

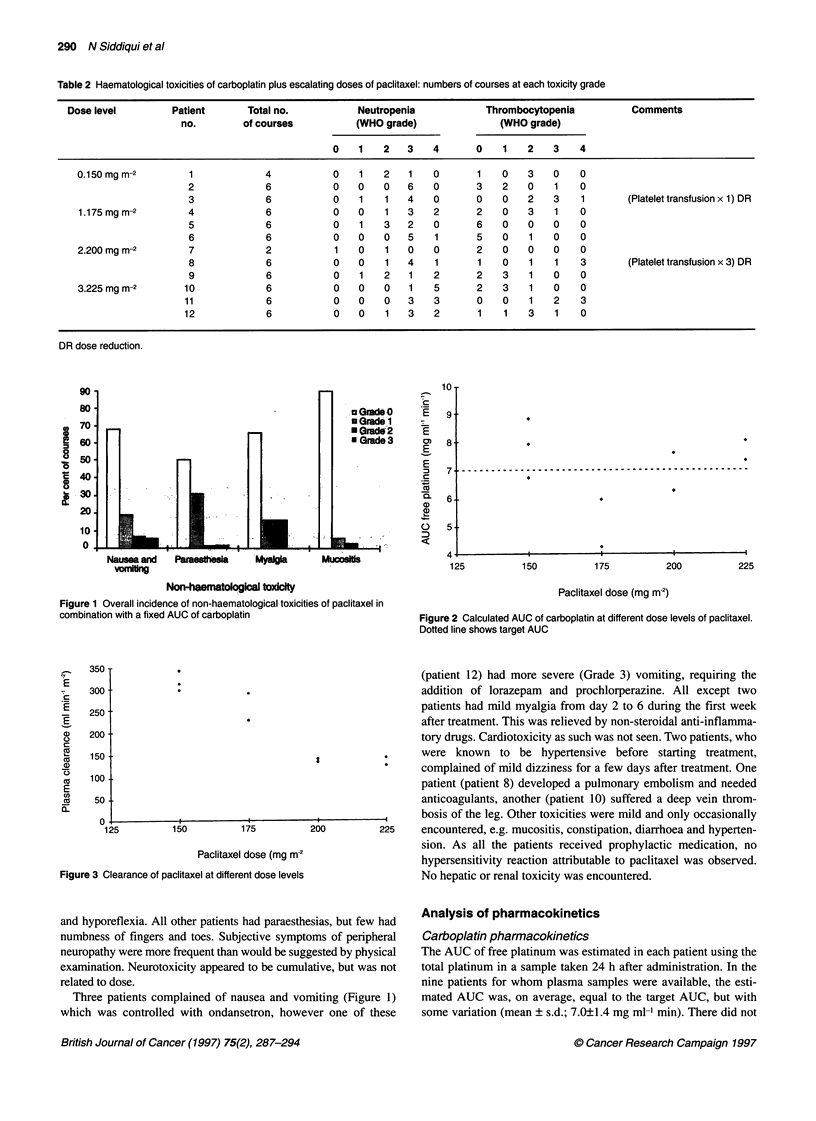

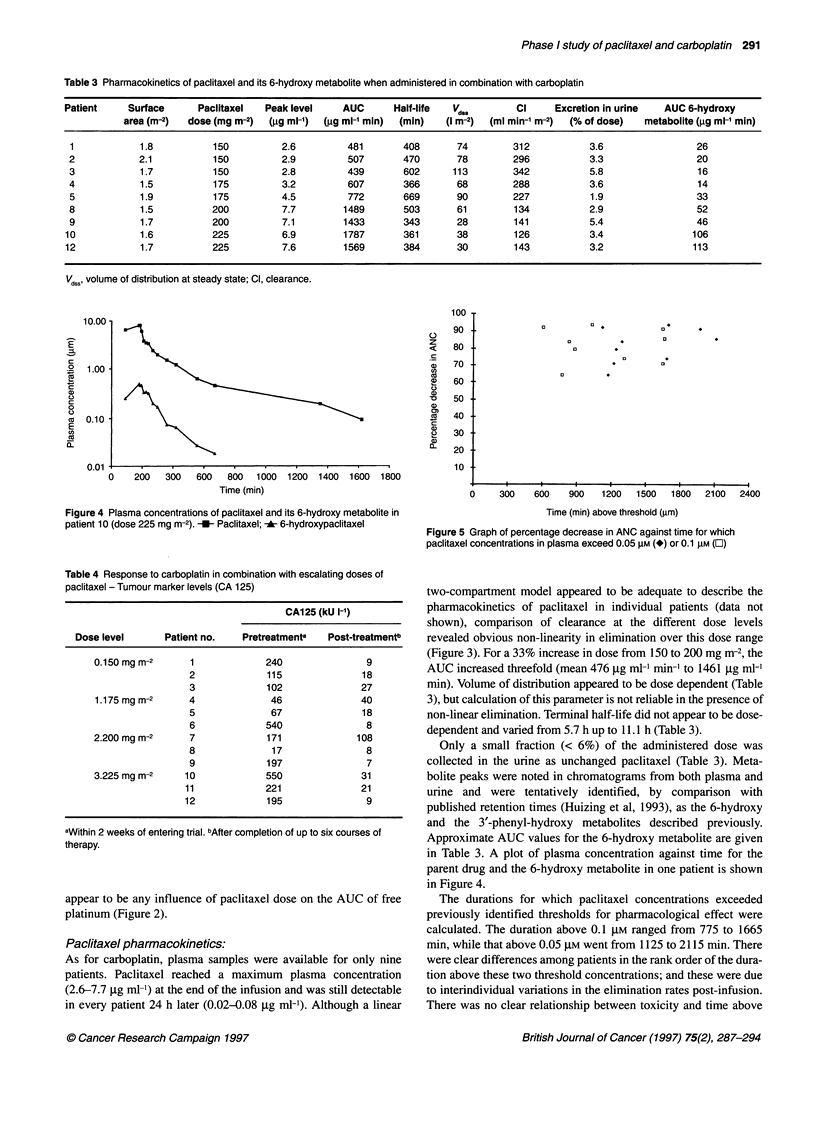

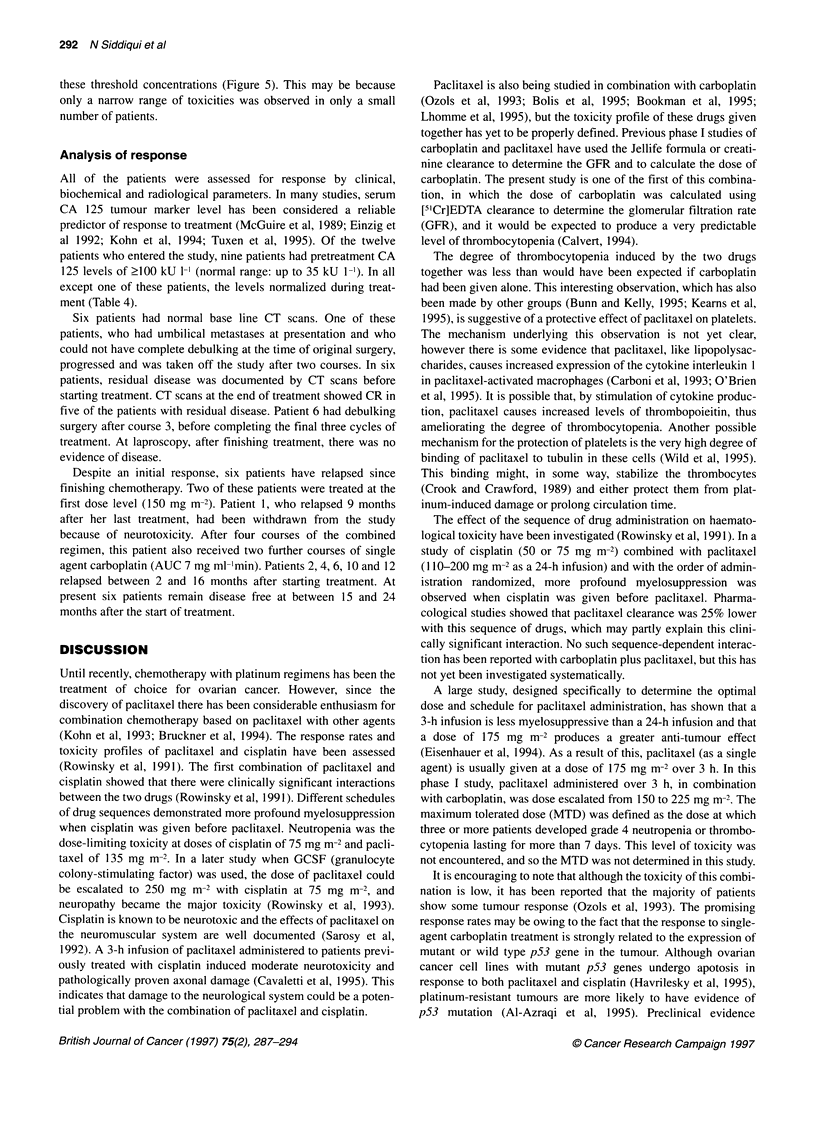

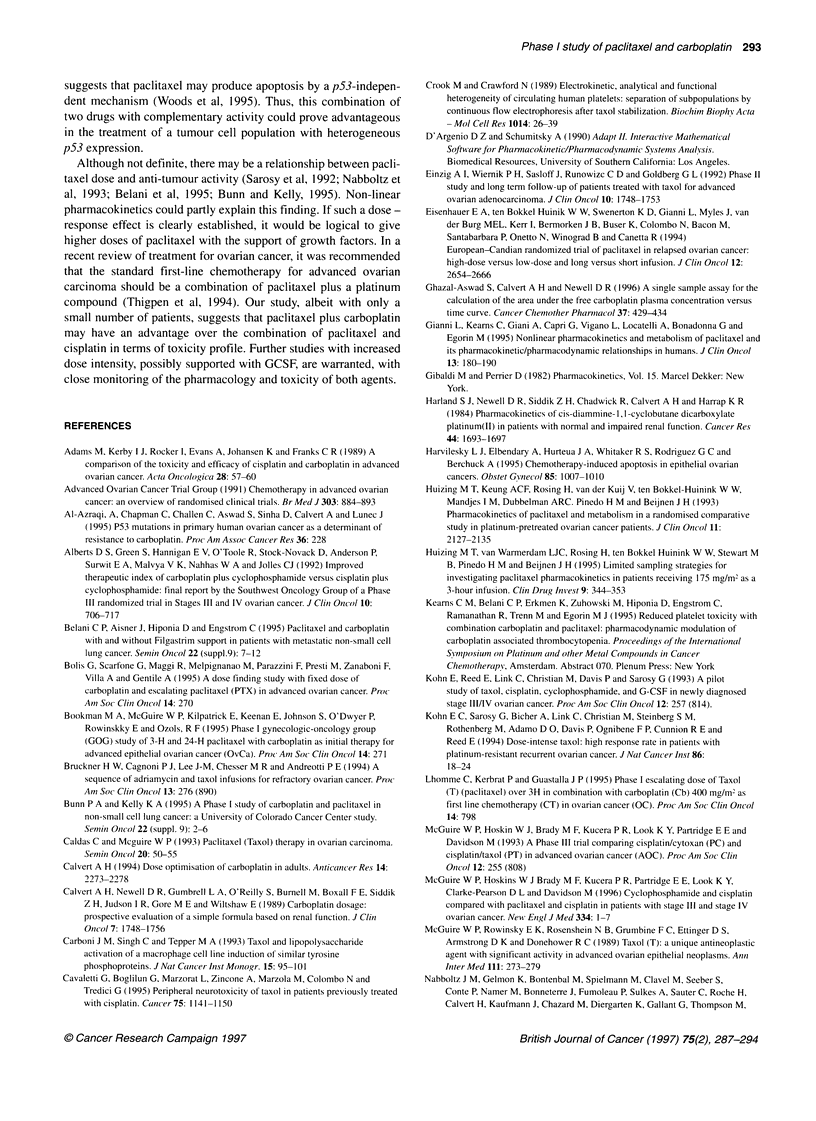

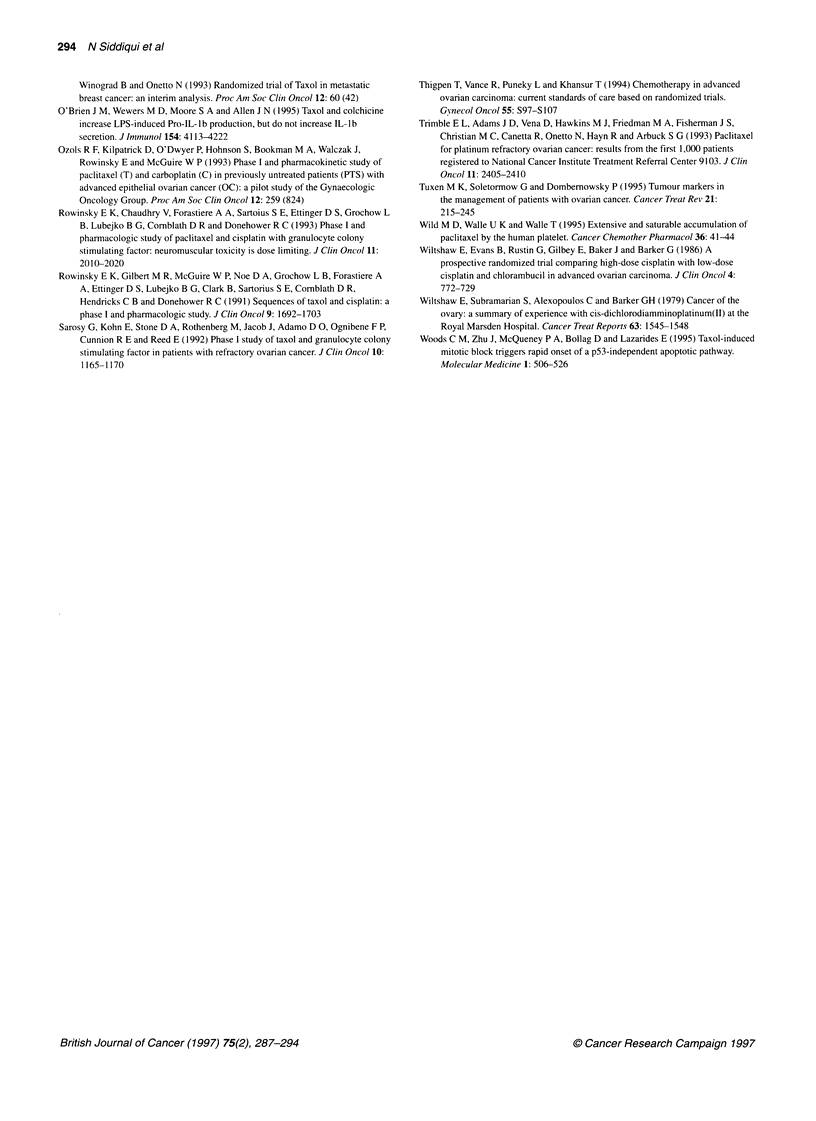


## References

[OCR_00892] Adams M., Kerby I. J., Rocker I., Evans A., Johansen K., Franks C. R. (1989). A comparison of the toxicity and efficacy of cisplatin and carboplatin in advanced ovarian cancer. The Swons Gynaecological Cancer Group.. Acta Oncol.

[OCR_00949] Caldas C., McGuire W. P. (1993). Paclitaxel (Taxol) therapy in ovarian carcinoma.. Semin Oncol.

[OCR_00951] Calvert A. H., Newell D. R., Gumbrell L. A., O'Reilly S., Burnell M., Boxall F. E., Siddik Z. H., Judson I. R., Gore M. E., Wiltshaw E. (1989). Carboplatin dosage: prospective evaluation of a simple formula based on renal function.. J Clin Oncol.

[OCR_00963] Cavaletti G., Bogliun G., Marzorati L., Zincone A., Marzola M., Colombo N., Tredici G. (1995). Peripheral neurotoxicity of taxol in patients previously treated with cisplatin.. Cancer.

[OCR_00970] Crook M., Crawford N. (1989). Electrokinetic, analytical and functional heterogeneity of circulating human platelets: separation of subpopulations by continuous flow electrophoresis after taxol stabilization.. Biochim Biophys Acta.

[OCR_00994] Ghazal-Aswad S., Calvert A. H., Newell D. R. (1996). A single-sample assay for the estimation of the area under the free carboplatin plasma concentration versus time curve.. Cancer Chemother Pharmacol.

[OCR_00999] Gianni L., Kearns C. M., Giani A., Capri G., Viganó L., Lacatelli A., Bonadonna G., Egorin M. J. (1995). Nonlinear pharmacokinetics and metabolism of paclitaxel and its pharmacokinetic/pharmacodynamic relationships in humans.. J Clin Oncol.

[OCR_01009] Harland S. J., Newell D. R., Siddik Z. H., Chadwick R., Calvert A. H., Harrap K. R. (1984). Pharmacokinetics of cis-diammine-1,1-cyclobutane dicarboxylate platinum(II) in patients with normal and impaired renal function.. Cancer Res.

[OCR_01018] Havrilesky L. J., Elbendary A., Hurteau J. A., Whitaker R. S., Rodriguez G. C., Berchuck A. (1995). Chemotherapy-induced apoptosis in epithelial ovarian cancers.. Obstet Gynecol.

[OCR_01025] Huizing M. T., Keung A. C., Rosing H., van der Kuij V., ten Bokkel Huinink W. W., Mandjes I. M., Dubbelman A. C., Pinedo H. M., Beijnen J. H. (1993). Pharmacokinetics of paclitaxel and metabolites in a randomized comparative study in platinum-pretreated ovarian cancer patients.. J Clin Oncol.

[OCR_01078] McGuire W. P., Rowinsky E. K., Rosenshein N. B., Grumbine F. C., Ettinger D. S., Armstrong D. K., Donehower R. C. (1989). Taxol: a unique antineoplastic agent with significant activity in advanced ovarian epithelial neoplasms.. Ann Intern Med.

[OCR_01096] O'Brien J. M., Wewers M. D., Moore S. A., Allen J. N. (1995). Taxol and colchicine increase LPS-induced pro-IL-1 beta production, but do not increase IL-1 beta secretion. A role for microtubules in the regulation of IL-1 beta production.. J Immunol.

[OCR_01102] Rowinsky E. K., Chaudhry V., Forastiere A. A., Sartorius S. E., Ettinger D. S., Grochow L. B., Lubejko B. G., Cornblath D. R., Donehower R. C. (1993). Phase I and pharmacologic study of paclitaxel and cisplatin with granulocyte colony-stimulating factor: neuromuscular toxicity is dose-limiting.. J Clin Oncol.

[OCR_01116] Rowinsky E. K., Gilbert M. R., McGuire W. P., Noe D. A., Grochow L. B., Forastiere A. A., Ettinger D. S., Lubejko B. G., Clark B., Sartorius S. E. (1991). Sequences of taxol and cisplatin: a phase I and pharmacologic study.. J Clin Oncol.

[OCR_01121] Sarosy G., Kohn E., Stone D. A., Rothenberg M., Jacob J., Adamo D. O., Ognibene F. P., Cunnion R. E., Reed E. (1992). Phase I study of taxol and granulocyte colony-stimulating factor in patients with refractory ovarian cancer.. J Clin Oncol.

[OCR_01129] Thigpen T., Vance R., Puneky L., Khansur T. (1994). Chemotherapy in advanced ovarian carcinoma: current standards of care based on randomized trials.. Gynecol Oncol.

[OCR_01134] Trimble E. L., Adams J. D., Vena D., Hawkins M. J., Friedman M. A., Fisherman J. S., Christian M. C., Canetta R., Onetto N., Hayn R. (1993). Paclitaxel for platinum-refractory ovarian cancer: results from the first 1,000 patients registered to National Cancer Institute Treatment Referral Center 9103.. J Clin Oncol.

[OCR_01140] Tuxen M. K., Sölétormos G., Dombernowsky P. (1995). Tumor markers in the management of patients with ovarian cancer.. Cancer Treat Rev.

[OCR_01145] Wild M. D., Walle U. K., Walle T. (1995). Extensive and saturable accumulation of paclitaxel by the human platelet.. Cancer Chemother Pharmacol.

[OCR_01148] Wiltshaw E., Evans B., Rustin G., Gilbey E., Baker J., Barker G. (1986). A prospective randomized trial comparing high-dose cisplatin with low-dose cisplatin and chlorambucil in advanced ovarian carcinoma.. J Clin Oncol.

[OCR_01154] Wiltshaw E., Subramarian S., Alexopoulos C., Barker G. H. (1979). Cancer of the ovary: a summary of experience with cis-dichlorodiammineplatinum(II) at the Royal Marsden Hospital.. Cancer Treat Rep.

[OCR_01159] Woods C. M., Zhu J., McQueney P. A., Bollag D., Lazarides E. (1995). Taxol-induced mitotic block triggers rapid onset of a p53-independent apoptotic pathway.. Mol Med.

